# A scoping review of service user experiences of potential harm in early intervention in psychosis services in the UK

**DOI:** 10.1111/bjc.70057

**Published:** 2026-04-16

**Authors:** Georgie Hudson, India Francis‐Crossley, Craig S. Mackie, Rachel Hiller, James B. Kirkbride, Claire Powell

**Affiliations:** ^1^ Division of Psychiatry University College London London UK; ^2^ Norfolk and Suffolk Foundation Trust, Early Intervention in Psychosis Services Norwich UK; ^3^ Clinical, Educational, and Health Psychology University College London London UK; ^4^ Population, Policy & Practice University College London London UK

**Keywords:** early intervention for psychosis, first‐episode psychosis, harm reduction, qualitative, scoping review, trauma

## Abstract

**Objectives:**

Early intervention in psychosis (EIP) services provide care for individuals experiencing first‐episode psychosis and are considered both clinically and cost‐effective. While many have positive experiences of EIP, there have also been reports of perceived harm. This scoping review synthesized qualitative evidence on service user perspectives of potential harm in EIP services in the UK, to consider how services may continue to improve practice to meet the needs of their service users.

**Methods:**

We conducted a scoping review following a pre‐registered protocol. Systematic searches were performed in PsycINFO, Web of Science Core Collection, Scopus, Embase, CINAHL Plus and Medline on 9th October 2024, and updated searches on 12th January 2026. We included peer‐reviewed qualitative studies exploring service user experiences in EIP services in the UK. Data on reported harms from eligible studies were thematically analysed.

**Results:**

Thirteen studies met inclusion criteria. We identified four main themes: (1) Intersection of stigma and lack of cultural awareness; (2) Inappropriate or inadequate support; (3) Poor relationships and communication; and (4) Challenges in the discharge process.

**Conclusions:**

While EIP services offer the current gold standard of care for first‐episode psychosis, our review highlighted some areas where harm was perceived to have arisen. Such information presents potential targets for service improvement. Strengthening communication and enhancing information sharing may help mitigate potential harms and optimize service user experiences. Further research to quantify the prevalence of harms within EIP services is needed to inform best practices and ensure equitable, person‐centred care.

**Practitioner points**

- Prioritize staff continuity and implement trauma‐sensitive handovers: High staff turnover forces service users to repeatedly share potentially traumatic personal stories, causing distress and diluting the quality of information shared. When staff changes are unavoidable, ensure thorough review of medical records and verification with service users rather than asking them to repeat their experiences.
- Enhance discharge planning with structured communication: Provide service users with regular transparent reviews of their progress, sufficient advance warning of discharge, and clear information about what to expect and when. Implement robust information‐sharing protocols between services to prevent service users from having to re‐explain their histories to new providers.
- Address cultural and spiritual needs proactively: Staff should develop knowledge and understanding of service users' cultural and spiritual needs to avoid misinterpreting culturally appropriate behaviours as symptoms of psychosis. Consider offering training on cultural competency and involving spiritual care representatives where appropriate.
- Improve transparency in care coordination: Clearly communicate staff availability, scheduling changes and care coordinator absences in advance to prevent distress. Ensure appropriate coverage arrangements are in place and communicated to service users when their primary contact is unavailable.

## INTRODUCTION

### Background

Early intervention in psychosis (EIP) services were introduced to the National Health Service (NHS) in 1999 (Department of Health, 1999) and have been nationally mandated in England since 2001 (Department of Health, 2001). EIP services aim to treat and support people experiencing a first episode of psychosis (FEP) for up to three years (NHS England, 2023) by providing NICE‐recommended interventions for care, including cognitive behavioural therapy, family intervention, and, if appropriate, clozapine (NICE, 2015). EIP services are made up of multidisciplinary teams that aim to provide timely, early psychological and pharmacological interventions, and support the service user and their families in order to enhance positive outcomes after the first onset of psychosis (Neale & Kinnair, 2017; NHS England, 2023). UK EIP services are designed around eight quality statements, including starting treatment within two weeks of referral, offering physical health interventions, and education and employment support (NICE, 2015), with recommendations that care co‐ordinators' caseloads not exceed 15 (Fern et al., 2025).

People with psychosis have higher rates of traumatic experiences (de Vries et al., 2019) and trauma‐related diagnoses such as post‐traumatic stress disorder (Hardy & Mueser, 2017) than the general population. A study of 100 early psychosis service users found that 82% had reported some exposure to childhood trauma (Duhig et al., 2015). This increased risk also extends to adulthood trauma. For example, a meta‐analysis of 27 studies found that adults with a psychotic disorder had a median prevalence of 66% for violent victimization, 39% for nonviolent victimization, and 27% for sexual victimization (de Vries et al., 2019). A prior history of trauma and adversity has also been shown to predict poorer outcomes, including reduced symptomatic remission and economic or educational inactivity, in EIP services in the United States (Jones et al., 2019). Although the causal basis of this relationship remains debated (Jauhar et al., 2022), trauma is strongly predictive of psychotic experiences, with a dose–response relationship and the cessation of trauma predicting the cessation of psychotic experiences (Kelleher et al., 2012).

EIP service users may be vulnerable to iatrogenic harm (inadvertent psychological, social or physical harm that arises from the process of treatment) (Markham, 2018) during their mental healthcare. Iatrogenic harm, which can also be described as ‘potential harm’ or ‘harm experienced in care’, is a well‐established concept in the context of physical and public health (Ashe, 2021; Carson‐Stevens et al., 2015; Christakis, 2009), yet remains under‐investigated in mental health services (Averill et al., 2023), although this has been more widely investigated with respect to harms perceived to be caused by medication, diagnosis or detention under the Mental Health Act (Aves, 2024). Iatrogenic harm can result in, or contribute to, adverse outcomes for service users, including feelings of shame and worthlessness, as well as suicidality (Aves, 2024). To the best of our knowledge, experiences and the frequency of iatrogenic harm in EIP services have never been formally assessed; however, it has been estimated in other mental health services. A scoping review of iatrogenic harm in mental health social care services in England found that social and psychological harm can be caused in service users by factors including poor relationships, discrimination, fragmented systems and poor support (Carr et al., 2023). A survey of 44 service users of mental health social care services found that all (100%) of the surveyed participants reported experiencing at least one form of iatrogenic harm in their care (Carr et al., 2025).

The high prevalence of trauma in service users with FEP may leave EIP service users in the United Kingdom vulnerable to iatrogenic harm. This means that further investigation is required to understand the current evidence base about potential harms experienced during EIP care in the UK.

### Aim

In this scoping review, we aimed to review the qualitativpeer‐reviewed literature about potential harms experienced by service users in the context of receiving EIP service care for FEP in the United Kingdom.

## METHODS

The design of the scoping review followed Arksey and O'Malley's (2005) methods, and used a systematic approach based on the Preferred Reporting Items for Systematic Reviews and Meta‐Analyses Extension for Scoping Reviews (PRISMA‐ScR) (Tricco et al., 2018). We pre‐registered the protocol on OSF (Hudson et al., 2024).

### Lived experience involvement

This scoping review incorporated the perspectives of those with lived experience. A lived experience advisory group (LEAG), comprising six members with experiences of psychosis, was involved in the data interpretation. The findings and preliminary themes of the review were discussed by the LEAG in two meetings, and their comments were incorporated into the final themes and the discussion section of this paper. Two members of the LEAG, including CSM, commented on and revised the manuscript.

### Search strategy

We used free‐text and medical subject heading (MeSH) terms to search six electronic databases: PsycINFO, Web of Science Core Collection, Scopus, Embase, CINAHL Plus and Medline. We used search terms related to four groups: qualitative, service user experiences, early intervention and psychosis (Supporting Information). The original search was conducted on the 9th October 2024 and re‐run on the 12th January 2026 to identify all papers published up to the 31st December 2025.

#### Inclusion criteria

Inclusion criteria required the papers to be: (1) qualitative or mixed methods empirical primary research; (2) peer‐reviewed articles; (3) include participants aged between 14 and 65 (the age group EIP services in the UK treat); (4) investigating service users' views and experiences of standard care in EIP services; (5) located in the United Kingdom; (6) published between 2000 and 2024 (as EIP services started in the NHS in September 1999 [Department of Health, 1999]).

### Citation screening

We imported the search results into Covidence (Veritas Health Innovation, 2024) for de‐duplication prior to screening. The first 10% of titles/abstracts were double screened by two reviewers (GH and IFC) with any disagreements resolved by consensus discussion and consulting with a third reviewer (CP) if necessary. Inter‐rater reliability (IRR) was assessed using Cohen's kappa (McHugh, 2012). It was determined a priori that if IRR met or exceeded a kappa value of .80 (indicating strong levels of agreement), the remaining titles/abstracts would be screened for inclusion by one reviewer. If IRR was below a kappa value of .80, the second reviewer would screen an additional 10% of titles/abstracts, and IRR re‐assessed. This process would be repeated until IRR reached a kappa value of .80, at which point the remaining titles/abstracts would be screened for inclusion by one reviewer (GH). The same process was applied again to the screening of full texts.

### Data extraction

From included studies, GH extracted: author(s); year of publication; study location study sample and population; study design and methodology; outcome measures; qualitative data on service user experiences of harm in EIP services. Only data extracts in the text labelled as ‘results’ or ‘findings’ were extracted, including verbatim quotations, consistent with recommended methodology (Dixon‐Woods, 2011; Thomas & Harden, 2008).

### Quality appraisal

The quality of included studies was established using the CASP Qualitative checklist (Critical Appraisal Skills Programme, 2024). This checklist provides a list of 10 items which should be included, and each item is rated as ‘yes’, ‘no’ or ‘can't tell’. As scoping reviews include a wide range of literature to understand a broad overview of the topic, no study was excluded based on quality, but study quality is reported in the findings.

### Strategy for data synthesis

Data were synthesized using Arksey & O'Malley's (2005) approach and utilized a ‘data charting form’ to record key information about each study, including methodology and results. Arksey & O'Malley's (2005) five stages are: (1) identifying the research question; (2) identifying relevant studies; (3) study selection; (4) charting the data; (5) collating, summarizing and reporting the results.

Extracted qualitative data were thematically synthesized using thematic analysis to develop an understanding of harm in EIP services. This involved three stages, which at points overlapped: (1) coding of text; (2) organization of codes to develop descriptive themes; and (3) development of analytical themes (Thomas & Harden, 2008). In stage 1, each line of text was inductively coded to develop an understanding of the content and meaning of the text. These codes were then grouped into themes by looking at their similarities and differences. The final stage involved creating a thematic map of the harms service users have experienced in EIP services.

Once the final themes were established, they were inductively mapped onto levels by which these harms could manifest. This was based on Bronfenbrenner's ecological systems theory and the analysis was conducted from an ecological perspective (Bronfenbrenner, 2000; McLeroy et al., 1988), which posits that interventions must target both individual and social factors to be effective, and that the multiple levels are interactive and reinforcing (Golden & Earp, 2012).

### Deviations from protocol

Due to methods that emerged as redundant during data synthesis, we deviated from the protocol (Hudson et al., 2024), and synthesized the data using thematic analysis, rather than framework analysis.

## RESULTS

Our initial search identified 1741 records from our database searches. Following de‐duplication, we screened 829 items by title and abstract (IRR = .87). We retrieved the full texts of 52 of these records, which we screened against inclusion criteria (IRR = .80). A total of 12 papers met our inclusion criteria, and an additional one was identified during updated searches, with a total of 13 papers included in the review (Figure 1).

**FIGURE 1:**
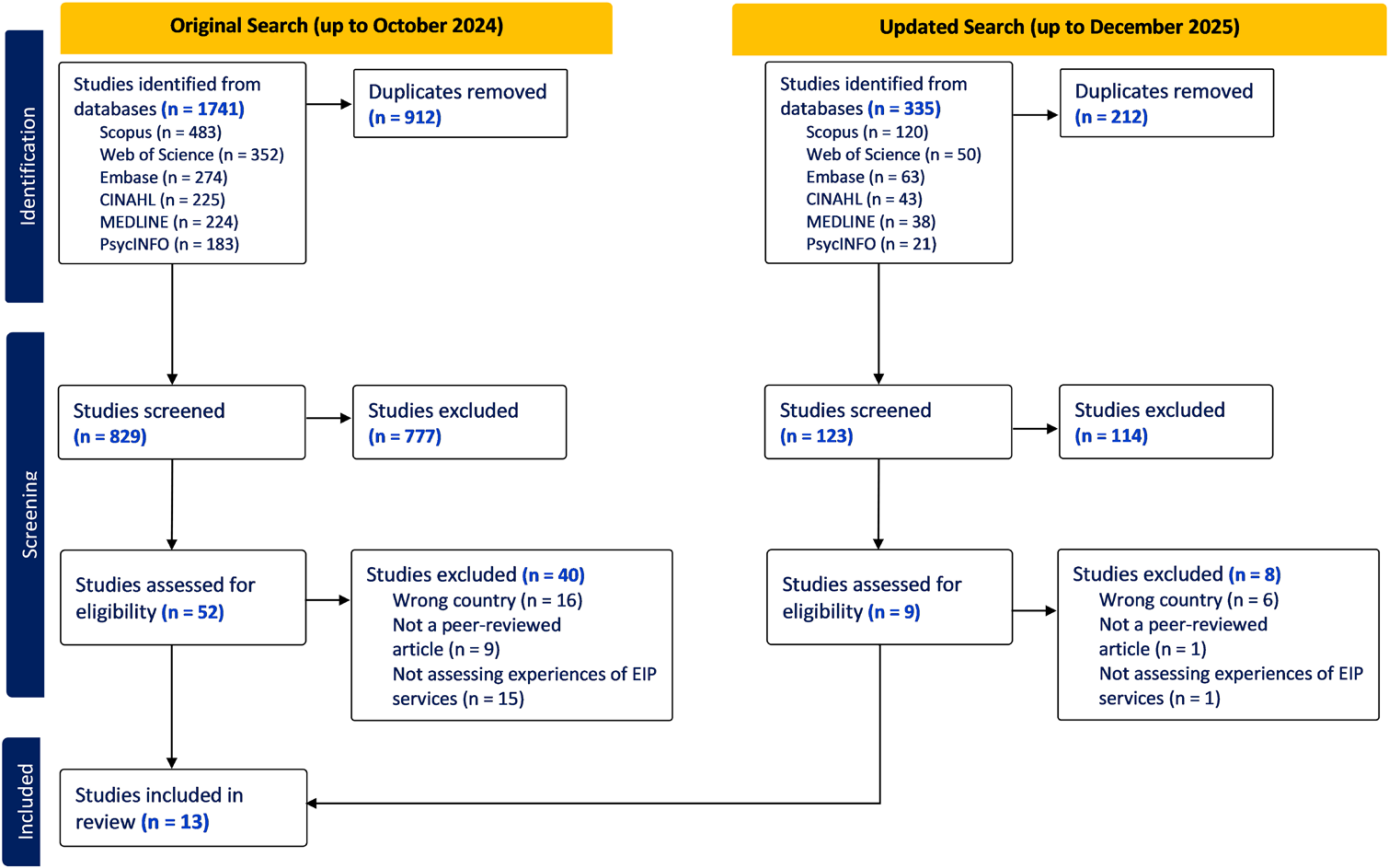
PRISMA diagram which details the number of records at each stage of the review process, from initial identification of records through to final study selection, including the updated search (conducted in January 2026).

### Overview of studies

Included papers were published from 2004 to 2025 (Table 1). Two papers (15%) employed mixed‐methods (Lee et al., 2023; Sakaria et al., 2024), with the remainder (85%) using solely qualitative methods (Allard et al., 2018; Das et al., 2014; Harris et al., 2012; Islam et al., 2015; Lester et al., 2011, 2012; Loughlin et al., 2019; Mankiewicz et al., 2018; O'Toole et al., 2004; Rickett et al., 2025; Watkins et al., 2018). Three of the 13 included papers (23%) reported no harms or negative experiences of EIP service users (Lee et al., 2023; O'Toole et al., 2004; Sakaria et al., 2024), and none of the included papers focused solely on assessing negative experiences or harms in EIP services.

**TABLE 1:** Summary of studies included in the review.

References	Study aims	Population & study location	Methodology	Participant information	Key findings
1. Allard et al. (2018)	To explore carers' and service users' experiences of UK EIP services	Participants were recruited from one site within the National EDEN multi‐site study	Service users nominated carers to be interviewed Interviews were analysed thematically, drawing on grounded theory and phenomenological analysis	32 participants (16 service users & 18 corresponding carers) *Service users*: Mean age: 22, range: 19–33 years Gender: 11 male (69%), 5 female (31%) Ethnicity: not reported *Carers*: Mean age: 55 years, range: 34–81 Gender: 15 female (83%) Relationship to service user: mother, father, stepmother, female partner, grandmother, grandfather Ethnicity: not reported	Two service users reported experiences that were not positive, with the EIP service unable to support the service user and establish a positive relationship These service users remained symptomatic and were reported to be ‘guarded’ and ‘difficult for staff and researchers to engage’
2. Das et al. (2014)	To investigate EIP services users' attitudes to antipsychotic long‐acting injections (LAIs)	Service users from an EIP service serving an urban catchment area in the northwest of England were purposively sampled: Service users prescribed either a LAI or an oral antipsychotic. Service users who had opted not to take antipsychotic medication. Male and females. White service users and service users from ethnic minority groups	Face‐to‐face, semi‐structured interviews conducted. Analysed via grounded theory	11 service user participants Median age: 24, range: 18–40 years Gender: 6 male (55%) Ethnicity: 10 white (91%), 1 Asian (9%)	Three themes: (1) therapeutic alliance & psychiatrists' recommendation of antipsychotics; (2) patients' knowledge and beliefs; (3) patients' views of appropriateness of LAIs Several participants reported side effects including weight gain and sedation All participants not currently prescribed an LAI reported knowing little/nothing about this option. Some currently prescribed an LAI would have chosen this earlier, had it been offered
3. Harris et al. (2012)	To explore service‐users' experiences of an EIP service, its impact on their experience of psychosis and on their current life situation	Participants were recruited using purposive sampling from an NHS EIP service. Those with the longest time in the service were approached first	Qualitative interviews were conducted flexibly using an interview schedule and analysed using interpretative phenomenological analysis	Eight service users, who had been in the EIP service for between 2 years and 2 years and 11 months Mean age: 29, range: 21–37 years Gender: 5 male (63%), 3 female (37%) Ethnicity: 5 White British (63%), 2 White & Asian (25%), 1 White & Black Caribbean (13%)	Participants reported a pre‐existing stigma around mental health services with them representing power and control Participants felt stigmatized by being in an EIP service, causing shame and separation from people with other mental health problems
4. Islam et al. (2015)	To examine the cultural appropriateness, accessibility and acceptability of the EIP service in Birmingham in improving the experience of care and outcomes for Black and minority ethnic service users	Service users, carers, community and voluntary sector organizations, service commissioners, EIP services staff members and spiritual care representatives were recruited. These groups were included to discover discrepancies or similarities in opinions All participants were recruited from the EIP service in Birmingham, UK. Service users were current or past users	Focus group methodology, with each group having separate focus groups Analysed thematically and drawing on framework analysis	Overall, 66 participants included *Service users*: 22 participants Mean age: 22 years Gender: 11 female (50%), 11 male (50%) Ethnicity: 9 Pakistani (41%), 1 Bengali (5%), 3 African (14%), 8 Caribbean (36%), 1 Arab (5%) *Carers*: 11 participants Age: 3 aged 36–45; 4 aged 46–55; 3 aged 55–65 & 1 aged 66+ Gender: 8 female (73%), 3 male (27%) Ethnicity: 3 Pakistani (27%), 2 African (18%), 5 Caribbean (45%), 1 White British (1%) *Voluntary organization representatives* 6 participants Gender: 4 female (67%), 2 male (33%) No other demographic information provided	Service users felt they had no choice of the gender of care co‐ordinators There were concerns around the quality, reliability and competence of interpreters, as well as occurrences of inappropriate behaviour and confidentiality concerns High staff turnover was reported, causing anxiety when a staff member they were close to was replaced. This also caused service users to repeat their stories, which was traumatic and detrimental to their wellbeing Staff members were unable to provide emotional support to service users Staff showed a lack of knowledge and understanding around service users' cultural and spiritual needs
				*Commissioners* 10 participants No other demographic information provided *Staff members* 9 participants Gender: 5 female (56%), 4 male (44%) No other demographic information provided *Spiritual care representatives* 8 participants Gender: 5 male (63%), 3 female (37%) No other demographic information provided	
5. Lee et al. (2023)	To evaluate an existing social drop‐in facility within an EIP service. Including how it was used; by how many people; opinions of the service; and areas of improvement	Staff and service users at the EIP service at the Birmingham and Solihull Mental Health NHS Foundation Trust	Mixed methods Attendance figures and demographics collected One focus group with current service users and another with staff members held. Analysed via thematic analysis	Overall, 9 participants across the two focus groups *Service users*: 4 participants Gender: 3 male (75%), 1 female (25%) Average age: 29 years Average time with service 37 months *Staff members*: 5 participants No demographic information provided	43 service users attended over 13 weeks (35% of caseload) Six key themes: (1) Reflection; (2) Environment; (3) Emotional experience; (4) Recovery; (5) Activities; (6) Interactions No harms reported
6. Lester et al. (2011)	To describe the views over time of young people in EIP services, with a particular focus on relationships	Participants were recruited from the cohort of service users in the National EDEN study of early intervention services. Service users were recruited from five geographically diverse sites across England, using maximum variation sampling (based on age, sex and ethnicity)	Semi‐structured interviews with participants aged 14–35 were conducted within six months after admission to EIP and then within six months of discharge Analysed using Charmaz's constructivist grounded theory approach	63 participated in an initial interview and 36 (57%) took part in a follow‐up interview at discharge Sex: 24 male (66%), 10 female (28%) Race‐ethnicity: 25 White British (68%), 1 other White (3%), 1 Irish (3%), 3 Pakistani (8%), 2 Indian (6%), 1 Black Caribbean (3%), 1 Mixed race (3%) Mean age at admission: 21 years (Men) & 23 years (Women)	1/3 felt visits from staff were too frequent. Continued regular presence reminded them they were being viewed as a patient needing treatment, when they felt ready to resume their lives Due to staff turnover, service users had multiple key workers causing them to be forced to repeat personal stories and receive repetitive advice
7. Lester et al. (2012)	To explore service users' perspectives of EIP services and primary care at two time points, with a particular focus on transition	As per Lester et al. (2011), Participants were recruited from the cohort of service users in the National EDEN study of EIP services. Service users were recruited from Birmingham, Lancashire, Norfolk, Cambridge and Cornwall, using maximum variation sampling based on age, sex and ethnicity	Semi‐structured interviews were conducted within six months after admission to EIP and then within six months of discharge Analysed using Charmaz's constructivist grounded theory approach	63 participated in an initial interview and 21 (33%) took part in a follow‐up interview 3 years' later Mean age at 1st timepoint: 23 years, range: 18–33 Ethnicity: 16 White British (76%), 1 Pakistani (5%), 1 Asian‐Indian (5%), 1 Bangladeshi (5%), 1 Indian (5%), 1 Mixed‐white/black African (5%) Gender: 14 male (67%), 7 female (33%)	Discharge experiences were generally positive but around a quarter had difficult transitions. These included unexpected/abrupt endings and poor information sharing between services Resulted in service users feeling helpless, abandoned and passed on or over
8. Loughlin et al. (2019)	To investigate the barriers and facilitators of transition from EIP services to primary and secondary care services in the UK from the perspectives of service users	Participants were recruited from five EIP services and community mental health teams across North‐West England	Interviews were conducted and analysed using thematic analysis	15 service users who had been discharged to primary/secondary services Gender: 13 male (87%), 2 female (17%) No other demographic information provided	News of discharge caused sadness, distress and feeling abandoned in those who felt reliant on the service Service users weren't given sufficient warning of discharge, which caused fear of relapse, feeling destabilized, and lost Some had poor relationships with staff due to personal differences of inconsistent contact Service users didn't trust staff to manage their handover and chose to do this themselves or allow family to do so Poor relationships led to feeling wary about forming new relationships, as well as dissatisfaction towards mental health services generally Participants had not received clear information and felt unable to make plans Not formally introduced to new services, left to navigate a new system alone and forced to repeat their stories again
9. Mankiewicz et al. (2018)	To explore expectations and experiences of receiving cognitive behavioural therapy for psychosis (CBTp) among EIP clients with FEP	Service users from a London‐based EIP services, where individual CBTp is offered as a core intervention	Semi‐structured interviews were conducted and analysed using thematic analysis	9 service users Mean age: 24.88, range: 17–33 Gender: 5 male (56%), 4 female (44%) Ethnicity: 8 White British (89%), 1 Black British (11%)	Participants broadly had positive experiences, but several participants viewed CBTp as a weak and ineffective treatment
10. O'Toole et al. (2004)	To explore service users' experiences of an EIP to help inform future service planning and provision	Participants were recruited from an EIP service in Southwark, London with a diagnosis of schizophrenia or schizoaffective disorder	Used a focus group methodology, analysed via interpretative phenomenological analysis	12 service users Mean age: 29.08 years, range: 17–53 Gender: 8 male (67%), 4 female (33%) Ethnicity: 6 White (50%), 2 Asian (17%), 2 Mixed (17%), 2 African/Caribbean (17%)	Aspects deemed important were: a human approach, involvement in decision‐making, flexibility, high staffing levels, reduction of symptoms, increased independence and confidence, and providing a daily structure No harms reported
11. Rickett et al. (2025)	To explore the service user and carer journey through discharge from EIP and into the early post‐discharge period	Participants were recruited at, or shortly after, discharge from EIP from six Mental Health Trusts (MHTs) around England	Semi‐structured interviews were conducted and repeated with service users at a 6‐month follow up Data were analysed using thematic analysis	30 participants included (16 service users and 14 carers) *Service users* Mean age: 40 years, range: 28–64 Gender: 9 female (50%), 9 male (50%) Ethnicity: 11 White British (69%), 1 White and Black Caribbean (6%), 1 Mixed (6%), 1 White Polish (6%), 1 Black Caribbean (6%), 1 White Australian (6%) *Carers* Mean age: 46 years, range: 25–69 Gender: 9 female (64%), 5 male (36%) Ethnicity: 8 White British (57%), 2 Black British (14%), 1 Indian British (7%), 1 Asian (7%), 1 Bangladeshi (7%), 1 White Italian (7%)	Two key themes: approaching discharge; post‐discharge needs and challenges. Some service users described feelings of abandonment and expressed a wish for transitional support
12. Sakaria et al. (2024)	To understand the experiences of service users who accessed an EIP service during the Coronavirus pandemic and compare these to a pre‐pandemic study within the same service (Watkins et al., 2018)	Service users of an EIP service between March 2020–2021, covering wealthy rural and deprived urban populations in the north of England	Mixed methods Overall satisfaction with the service assessed via Likert scales in a questionnaire Interviews analysed via thematic analysis	18 service users completed the questionnaire, 6 (33%) undertook interviews No demographic information provided	Participants were broadly satisfied with all areas of the service. No overall dissatisfaction reported Participants felt safe and supported by staff No harms reported
13. Watkins et al. (2018)	To gather the perspectives of individuals accessing an EIP service in order to inform service development	Current service users of an EIP service serving both urban and rural populations	Individual interviews and one focus group conducted. Data were analysed using thematic analysis	16 service users (7 in a focus group and 9 individual interviews) Age range: 14–35 years No other demographic information provided	Participants reported unclear communication around when their care coordinator would be unavailable, causing distress. Service users reported inappropriate or non‐existent cover when their care coordinator was unavailable Irregular appointments and a lack of consistency and continuity of care caused distress Staff ‘sugar‐coated’ service users' progress, leading to frustration and disappointment when service users' expectations were not met, or a feeling of staff not understanding service users' experiences Staff normalizing symptoms or experiences caused conflict as service users felt minimized or invalidated

The number of participants included in these studies' qualitative analyses ranged from eight (Harris et al., 2012; Lee et al., 2023) to 66 (Islam et al., 2015) with a total of 289 participants across all studies (two studies reported on the same participant group, but data for these studies were extracted and analysed separately as they investigated different research questions). All studies that identified the specific location or service from which they recruited participants in the UK were based in England, with no studies identified from the devolved nations. Where detailed, study locations included Birmingham (Islam et al., 2015; Lee et al., 2023; Lester et al., 2011, 2012); Solihull (Islam et al., 2015); Lancashire, Norfolk, Cambridge and Cornwall (Lester et al., 2011, 2012); and London (Mankiewicz et al., 2018; O'Toole et al., 2004). Nine studies (69%) investigated only one location, with 3 (23%) investigating multiple locations across England (Lester et al., 2011, 2012; Loughlin et al., 2019). Six studies (46%) did not specify their exact location in the UK, but reported that participants were recruited from: (1) an NHS EIP service (Harris et al., 2012; Watkins et al., 2018); (2) North‐West England (Das et al., 2014; Loughlin et al., 2019); (3) one site within the National EDEN multi‐site study (Allard et al., 2018); or (4) six mental health trusts around England (Rickett et al., 2025).

### Quality appraisal

The included studies' quality was appraised using the CASP Qualitative Studies checklist (Critical Appraisal Skills Programme, 2024) (Table 2). Overall, included studies were generally of satisfactory quality, although only two studies were rated ‘yes’ in all categories (Loughlin et al., 2019; Mankiewicz et al., 2018). Most commonly, studies did not include sufficient information on the relationship between participants and researchers or did not provide adequate information on ethical issues.

**TABLE 2:** Quality appraisal of the included qualitative studies using the CASP checklist (Critical Appraisal Skills Programme, 2024).

Author (year)	Was there a clear statement of the aims of the research?	Is a qualitative methodology appropriate?	Was the research design appropriate to address the aims of the research?	Was the recruitment strategy appropriate to the aims of the research?	Was the data collected in a way that addressed the research issue?	Has the relationship between researcher and participants been adequately considered?	Have ethical issues been taken into consideration?^a^	Was the data analysis sufficiently rigorous?	Is there a clear statement of findings?	Is the research valuable?
AIlard (2018)	✗	✓	?	?	?	✗	?	✓	✓	✓
Das (2014)	✓	✓	✓	✓	✓	?	✓	✓	✓	✓
Harris (2012)	✓	✓	✓	✓	?	✓	✓	✓	✓	✓
Islam (2015)	✓	✓	✓	✓	✓	✗	✓	✓	✓	?
Lee (2023)	✓	✓	✓	✓	?	✗	?	✓	✓	?
Lester (2011)	✓	✓	✓	?	✓	✗	?	✓	✓	✓
Lester (2012)	✓	✓	✓	✓	✓	?	?	✓	✓	✓
Loughlin (2019)	✓	✓	✓	✓	✓	✓	✓	✓	✓	✓
Mankiewicz (2018)	✓	✓	✓	✓	✓	✓	✓	✓	✓	✓
O'Toole (2004)	✗	✓	✓	✓	?	✗	?	✓	✓	✓
Rickett (2025)	✓	✓	✓	✓	✓	?	✓	✓	✓	✓
Sakaria (2024)	✓	✓	✓	✓	✓	✗	?	?	✓	✓
Watkins (2018)	✗	✓	✓	?	✓	✓	✓	✓	✓	✓

*Note*: Response options: yes ✓; no ✗; or unclear ?

^a^ This question was marked as ‘unclear (?)’ if no formal ethical approval reported but no ethical concerns identified.

### Findings

We organized our findings into four key themes on harm to service users that was perceived by service users, carers and other stakeholders to be caused by EIP services. These four themes were: (1) Intersection of stigma and lack of cultural awareness; (2) Inappropriate or inadequate support; (3) Poor relationships and communication; and (4) Challenges in the discharge process. These four themes described harms that could be caused at systemic, cultural and relational levels (see Figure 2; adapted from Bronfenbrenner [2000]).

**FIGURE 2:**
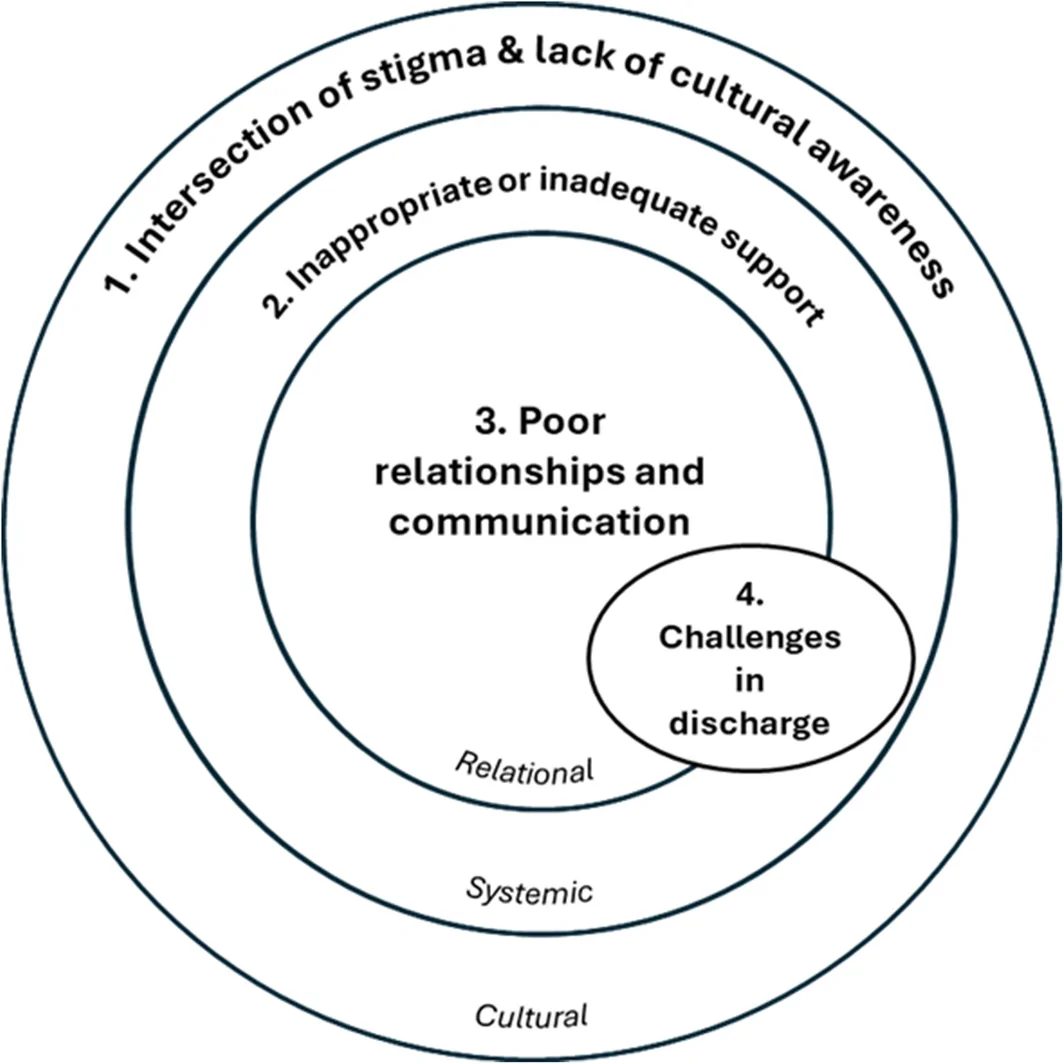
Overview of themes extracted from the qualitative data on harm in EIP services and how they map onto relational, systemic and cultural levels at which they may occur. Adapted from Bronfenbrenner's ecological systems theory (Bronfenbrenner, 2000).

### Intersection of stigma and lack of cultural awareness

Across three papers (Harris et al., 2012; Islam et al., 2015; Lester et al., 2011), we found evidence that organizational cultures and practices could create environments where service users felt stigmatized; this theme occurred at the cultural level of Bronfenbrenner's model (Figure 2). Additionally, some service users also reported that EIP services did not take their cultural or spiritual needs into account. In Islam et al.'s (2015) study of 22 service users, which investigated the cultural appropriateness of an EIP service in Birmingham, England from the perspective of service users from minoritised ethnicities, some participants reported that EIP service staff lacked knowledge and understanding of service users' cultural and spiritual needs, sometimes interpreting them as symptoms of psychosis:

We had… a Baptismal Service, and quite a number of the staff came, and one of the service users suddenly spoke in tongues…The result of that was a bit of a shock wave, and his psychiatrist was thinking of upping his medication… So of course I was then, able to speak to that particular consultant psychiatrist. (Male spiritual care representative) (Islam et al., 2015).

In two papers (Harris et al., 2012; Lester et al., 2011), service users reported stigma around mental health services, and of being viewed and labelled only as a patient, even after feeling they were ready to be ‘free from everything and move on’ (Lester et al., 2011). For example, participants in Harris et al.'s (2012) study felt particularly stigmatized as service users of an EIP service, causing them to feel shame and separation:

The fact that it's not known and it's this little… specialised service … and you're sort of in this group and you're not in sort of the mainstream I guess. (Female service user, age 29) (Harris et al., 2012).

This feeling of separation from other ‘mainstream’ mental health services was thought by service users to result from the stigma of psychosis still existing in society, despite other mental health conditions having become more accepted (Harris et al., 2012). Participants in Harris et al.'s study ‘suggested a pre‐existing stigma surrounding traditional mental health services, with them representing power and control over people. This stigma was seen as being projected onto the EIP service’. This stigma resulted in some service users reporting feelings of ‘shame and separation’ (Harris et al., 2012) from other mental health service users.

#### Inappropriate or inadequate support

Across five papers (Islam et al., 2015; Lester et al., 2011; Loughlin et al., 2019; Rickett et al., 2025; Watkins et al., 2018), service users reported experiencing levels of care that failed to meet their needs, which occurred at the systemic level according to Bronfenbrenner's model (Figure 2). A recurring sub‐theme was a lack of staff consistency and continuity in EIP services. For example, participants reported having different key workers or care co‐ordinators during their care due to high staff turnover, resulting in service users being forced to repeat their personal and potentially traumatic stories multiple times, often receiving repetitive advice in return (Islam et al., 2015; Lester et al., 2011; Rickett et al., 2025; Watkins et al., 2018).

it's a bit annoying, I think, especially when you are not feeling good mentally, when you're having to repeat yourself and tell your story time and time again (Rickett et al., 2025).

High staff turnover led to service users ‘continually having to get to know different people and to tell my story, and it takes a whole load of time to build up trust in someone’ (Lester et al., 2011), causing distress and anxiety (Islam et al., 2015; Lester et al., 2011; Watkins et al., 2018). Low staffing was also reported as a key barrier to access to care, as ‘If [my Care Coordinator] wasn't available I'd be stuck for like three weeks upset and waiting to talk to someone’ (Watkins et al., 2018). A participant in one study reported that ‘it's just a shame that I didn't get the full level of care… due to staffing’ (Loughlin et al., 2019). As well as having a consistent point of contact who could support them, service users valued regularly scheduled appointments, while a lack of care between appointments was distressing for service users (Watkins et al., 2018).

Included studies reported different service users experiencing both too little and too much support as potentially harmful. For approximately one third of 63 participants in one study (Lester et al., 2011), the degree of engagement with the EIP service was deemed too intense, with home visits from staff members seen as intrusive as ‘it's not nice having somebody come in all the time, because it constantly reminds me that you're suffering from an illness’ (Lester et al., 2011). This was also related to high staff turnover, as different staff members ‘seem to ask the same questions all the time’, forcing service users to repeat their personal stories (Lester et al., 2011). However, two studies reported other service users also experienced a lack of support, particularly emotional support, from EIP service staff (Allard et al., 2018; Islam et al., 2015). Allard et al. (2018) briefly discussed service users' negative experiences of EIP services, with the study authors reporting that EIP services were unable to provide adequate support to two of 16 service users; however, no further information on specific negative experiences was provided. Islam et al. (2015) reported that while some service users sought out emotional support from their EIP services, this was not received; instead, service users were recommended to approach voluntary sector organizations for this.

A lack of clear information and choice over care was reported by some service users. Das et al.'s (2014) study of attitudes towards long‐acting injectable (LAI) antipsychotic medication found that of those service users who were not currently prescribed an LAI, they knew either little or nothing about this option, with some reporting they would have chosen an LAI earlier had they known it was available. The lack of choice over medication in some cases resulted in service users experiencing side effects from being prescribed medications that did not suit them, in addition to their psychotic symptoms not being alleviated (Das et al., 2014).

A lack of choice also extended to input into their care providers, with some service users reporting they had not been given a choice about the gender of their care coordinator (Islam et al., 2015). Loughlin et al. (2019) also reported a service user concern about ‘a lack of information, I'm not a mind‐reader’, which coincided with service users feeling unsure about their future. One service user appeared to have particularly negative experiences of EIP services, ‘describing it […] as “three wasted years”’ in an interviewer's reflective journal (Loughlin et al., 2019).

In addition to experiencing medication side effects and persistent psychotic symptoms, service users reported that receiving inappropriate or inadequate support—such as limited contact with the service—led to feelings of wariness and mistrust towards both the specific service and mental health services more broadly (Loughlin et al., 2019).

### Poor relationships and communication

Evidence from six papers (Allard et al., 2018; Das et al., 2014; Islam et al., 2015; Lester et al., 2011; Loughlin et al., 2019; Watkins et al., 2018) indicated that poor relationships and communication with staff members resulted in some service users experiencing distress, sadness, mistrust and a fear for their future. These experiences occurred in EIP services at a relational level according to Bronfenbrenner's model (Figure 2). Poor communication from staff to service users was associated with distress in service users, particularly in relation to not being made aware of staff unavailability, or scheduling changes:

Not being told that my Care Coordinator is off due to illness or whatever until afterwards […]caused me to have some problems (Watkins et al., 2018).

Loughlin et al. (2019) reported that some service users ‘did not have a good relationship with early intervention staff because of interpersonal differences’, leading to poor relationships with the staff members, and the service in general, as ‘he [keyworker] wasn't the right guy’. In some cases, these poor relationships led to service users feeling mistrustful of EIP service staff members in managing their care.

One study reported a lack of honest feedback from staff about service users' progress that resulted in service users feeling disappointed; ‘set[ting] them back on their recovery journey’ (Watkins et al., 2018). This was most frequently in reference to service users feeling that staff were exaggerating and over‐promising their progress, which led to false hope and disappointment when it transpired this was not the case, setting them back on their recovery journey:

When I've been sugar‐coated in sweetness […] I haven't had the honesty […] it's put me back a bit because I think I'm doing good […] in the long run I find actually I'm not doing good (Watkins et al., 2018).

Staff over‐promising progress, and attempting to normalize their symptoms and experiences, was interpreted by some service users as staff not understanding them and their experiences, leaving them feeling invalidated or minimized:

I've been told too many times in the last few weeks that I'm just having a bad day and it's a blip[…] and it's just like […] it's very frustrating because it's not just one bad day, it's a continuous bad day (Watkins et al., 2018).

### Challenges in the discharge process

Three studies reported harms caused by an inappropriate and poor discharge process, which occurred across systemic and relational levels according to Bronfenbrenner's model (Figure 2) (Lester et al., 2012; Loughlin et al., 2019; Rickett et al., 2025). Much of the data in this theme were extracted from Loughlin et al. (2019) and Rickett et al. (2025), who investigated service user experiences of discharge from EIP services. One challenge here included a lack of warning of an imminent discharge, leading to service users being left to navigate this information alone. This abrupt change caused them to fear for their future, in terms of fear of relapse, and their future mental health. Challenges in the discharge process also caused some service users to feel wary of the new service to which they were being discharged (Loughlin et al., 2019). Many participants in this study reported being discharged soon after being informed that it would be happening, which caused them to feel destabilized and lost:

Because it was so sudden… I'd not had the sit down… “look, this is what's gonna happen, this is how it's gonna work”… there should be… a middle ground like a stepping stone, to say “look, this is how it's going to work” and just give people a bit more information about it, before they just chuck them into another service (Loughlin et al., 2019).

Loughlin et al. (2019) reported that a lack of clear information from the service led some service users to feeling uncertain about their future care:

They always concentrate on the current, the present tense. It would have been nice to have a look into the future and say “right OK, in the future, this is gonna happen” (Loughlin et al., 2019).

A lack of joined up working and information sharing between the EIP services and the primary or other secondary care service that service users were discharged to resulted in some service users feeling abandoned, helpless, and ‘of being bereaved and a sense of loss’ (Rickett et al., 2025):

My CPN [community psychiatric nurse] turned round and went ‘oh good luck in the future. I might see you in the future’. And sort of hugged me in the street and then walked away. It was very strange. I knew it was going to happen but I wasn't given a confirmed date. A firm date would have been much better and a bit nicer than ‘oh good luck you've done it’ and that's it (Lester et al., 2012).

This lack of joined up working also caused some service users to feel frustrated and abandoned, left to navigate an unfamiliar, new system and service alone (Loughlin et al., 2019; Rickett et al., 2025). Those who reported poor interpersonal relationships with staff members were also more likely to mistrust staff members' abilities to successfully manage their handover, with some service users resorting to managing their discharge themselves (Loughlin et al., 2019). Planned handover meetings with CMHTs were reported to not always occur (‘they did try to meet together, but it just didn't work out’ [Rickett et al., 2025]).

Some service users reported that when approaching discharge from the EIP service, they still felt practically or emotionally dependent on the service, with the impending discharge resulting in sadness, distress and a feeling of abandonment:

It's [the news of discharge] like someone telling you your family have been in a car crash or something, that's the best way to explain it. Really, that's how horrible a feeling it is (Male service user) (Loughlin et al., 2019).

Some attempts by staff members to discuss discharge with service users were seen as ‘tokenistic and inauthentic’ (Rickett et al., 2025), as the service's ‘3‐year time limit forestalled any negotiation’ (Rickett et al., 2025). This resulted in some service users feeling disconnected from decision‐making and ‘pressured into discharge’ (Rickett et al., 2025).

When the decision is—“You're leaving us and you're going to the CMHT,” and there's no decision to be made and there's no choice—you can flannel and you can dress it up, but it is what it is. So, in a way, it was not particularly easy at all and, in fact, it was a very bad transition in my opinion (Rickett et al., 2025).

## DISCUSSION

### Principal findings

In this scoping review, we synthesized the published qualitative literature of how service users can experience potential harm during their EIP service care in the UK. To our knowledge, this is the first review to have explored this issue. We identified four key themes of how potential harm may arise: (1) intersection of stigma and lack of cultural awareness; (2) inappropriate or inadequate support; (3) poor relationships and communication; and (4) challenges in the discharge process. These harms manifested across cultural, systemic and relational levels of Bronfenbrenner's model (Figure 2) and had wide‐ranging consequences on service users, including distress, feeling abandoned or helpless, medication side effects, trauma and a mistrust of staff, EIP services and general mental health services. Our results could help signpost service commissioners, providers and managers to themes that could help guide and improve service delivery.

### Comparison with prior literature

The four themes we identified in this scoping review are similar to those found by a qualitative review of avoidable harm in mental health social care in England (Carr et al., 2023). The Carr et al. review also found that service users could be harmed by poor relationships and communication, poor information and involvement, poor support, staff discontinuity and discriminatory cultures (Carr et al., 2023). However, harm caused to service users by challenges in the discharge process was not a specific theme in the Carr et al. review, suggesting it may be a more relevant issue for service users in EIP services. Previous work has suggested that service users should be monitored for a minimum of one‐year after discharge from EIP services, to extend care continuity and reduce risk of relapse (Kam et al., 2015).

We also found that some service users felt that EIP services were particularly stigmatized services by the general population, causing them to feel a degree of separation from people using other mental health services (Harris et al., 2012).

Although not a direct finding, trauma‐informed care (TIC) may be a useful model to understand service user experiences of potential harm in EIP services. Based on the recognition of the widespread prevalence of trauma in people with psychosis, an international Delphi study was conducted to reach consensus on how TIC principles should be conceptualized in EIP services (Mitchell et al., 2021). TIC in psychosis is based on the knowledge and understanding of how trauma can affect people and includes principles such as an empathetic and non‐judgmental environment and adopting a person‐centred approach (Mitchell et al., 2021). TIC is designed so that staff are trained to assume that service users have experienced trauma and represents a change in thinking from ‘what is wrong with you?’ to ‘what happened to you?’ (Harris & Fallot, 2001; Sweeney et al., 2016). The Delphi study was set in the context of a Northern Ireland EIP service and so has relevance to the UK setting of our work, although the participants were international experts who had published first‐person accounts of psychosis (Mitchell et al., 2021). Panel members agreed that trauma‐informed training, knowledge and supervision for EIP staff was highly important and that TIC in EIP services will work to protect service users against ongoing harms and abuse.

In addition, TIC has been introduced in some mental healthcare settings in the UK, such as inpatient psychiatric care in the Tees, Esk and Wear Valleys NHS Foundation Trust (2023). TIC is a useful model for thinking about service reorganization, which may be particularly relevant in EIP care, given the prevalence of trauma experienced by people with psychosis. However, there are some issues in agreeing on what the optimum TIC model of care looks like, as well as an operational definition (Bendall et al., 2021). In some jurisdictions a TIC approach has been applied to EIP care. Australia is considered a global leader in trauma‐informed mental health care, with the Orygen Specialist Program providing mental health services to young people embedding TIC into its approach (Creamer, 2016). Although an international survey suggests a low rate of implementing trauma‐informed approaches in EIP services more generally (Wood et al., 2023). In the UK, EIP services are not currently required to adhere to TIC approaches in the national guidelines for EIP care. However, the extent to which the formal implementation of TIC in EIP services would reduce harms experienced by service users remains unknown.

Our findings could be used to map potential harms that may arise during EIP care onto the guidelines by Sweeney et al. (2022) for implementing trauma‐informed psychological therapy assessments. This survivor‐led study recommended that services focus on relationships, transition from systems to people, reflect on power and understand intersectionalities. These are similar to our review's suggestions that harm to service users can be reduced via improved staff‐service user communication, increased cultural understanding, and greater institutional and managerial support for staff.

### Implications and recommendations

In the UK, the NHS and its staff members are under acute pressures caused by high need for care and chronic underfunding after more than a decade of economic austerity. Despite this, there is evidence of the positive impact EIP services have for many service users with FEP (Craig et al., 2004; Dodgson et al., 2008; Garety et al., 2006; Singh, 2010); they are valued by service users (Lester et al., 2012) and remain the gold standard for treatment of FEP in England and many other nations (Correll et al., 2018; Salazar de Pablo et al., 2024). Our findings signpost possible areas where services could mitigate unintended harms to further improve care.

We found that harms to service users can result from problems across cultural, systemic and relational levels of care, potentially necessitating change across all three levels. For example, we found that challenges in discharge from EIP services—most pertinently a systemic and relational issue—can be particularly distressing to service users. Although discharge from EIP services is inevitable due to their three‐year care mandate (NHS England, 2023), our findings suggest that improvements in the discharge process are warranted. Based on our review, one recommendation would be that service users receive regular, transparent reviews as to their progress and sufficient warning of their upcoming discharge. This discussion around discharge may even begin at the point of intake to the service to ensure service users' expectations are clear from the beginning. This should be accompanied by the provision of clear information about what service users can expect, and at what time point during this process. Finally, the discharge process should incorporate high levels of information sharing between services so that service users are not required to repeat all information they had previously given to the EIP service.

Other themes we identified in this review were poor relationships and communication, and inappropriate or inadequate support, which had a negative impact on some service users due to high staff turnover and the lack of consistency this caused for service users – another systemic and relational issue. These themes particularly resonated with the LEAG consulted throughout the process of this review. They reported that in their experience of service use for psychosis, they frequently had to repeat their stories over again to new staff members and that each time they had to repeat their frequently traumatic stories, it was diluted until after a while they were almost telling a different story to the staff members. As repeating their stories can be traumatic and impact the quality of the information the service users report, we strongly recommend that systemic changes be made to reduce this. Ideally this would result from improving staff consistency and reducing turnover; however, we acknowledge that this may not always be feasible. Additionally, if service users experience changes in staff, we suggest that systems are put in place so that all members can ensure that the service users' medical records have accurate notes which are checked with the service users, and that new staff members familiarize themselves with the notes, rather than requesting the service user to repeat themselves. Artificial intelligence may be useful to summarize previously written notes for new staff members.

As a general recommendation for EIP services, our LEAG emphasized the importance of advocacy for service users and the importance of a whole family approach when treating psychosis. Advocacy could come from family members or friends, as well as external, formal sources. As people with FEP are often navigating an unfamiliar service while acutely unwell, having an external person explain their rights and providing a voice for them if they are unable to may help support and empower service users to ultimately advocate for themselves (Sunkel, 2012). Our lived experience advisors suggested that providing peer support to service users, as well as psychoeducation on mental health to both service users and those close to them may aid in this progress as well.

### Strengths and limitations

We employed a comprehensive search strategy, searching six databases. We searched and screened literature based on whether they investigated any experiences of EIP services in order to capture all relevant literature. This review also consulted with a LEAG of six members who have all experienced psychosis, to ensure the findings reflect those with lived experience of psychosis; however, they were not necessarily treated in EIP services due to the time at which they first experienced psychosis.

It is important to note that in this review we only extracted and analysed the data specifically describing harms. Many studies included in this review also included service user descriptions of good practice and positive experiences, so while we can conclude that some service users do experience harm during their care in EIP services, we do not claim that this occurs to all service users. Due to the qualitative focus of this scoping review, we did not aim to investigate the frequency of harms to service users, nor the percentage of service users in EIP services who experience harms. We recommend that future epidemiological work investigates the prevalence of these harms to understand the extent and context of these harms, as such evidence appears missing from the literature.

As is the case with most qualitative research, this review relied on studies with small sample sizes, limiting the generalisability of our findings. It is also possible that some studies suffer from sampling bias, as service users with particularly positive or negative experiences may be more likely to participate in a study investigating these experiences (McCambridge et al., 2014). This review focused on experiences within EIP services, and it was outside the scope to explore experiences in mental health services more broadly. However, it may be that the harms discussed here apply to broader mental health provision. This remains an important area of exploration.

Additionally, due to practicalities and the scope of this review, we were only able to search the published, peer‐reviewed literature. Future work should aim to investigate this topic further in grey literature, particularly focusing on reports of organizations, charities, and regulators, as well as service users' blogs and articles, although some of the aforementioned limitations are also likely to exist in some of these sources.

It is also important to note that some of the studies included in the review may be somewhat outdated, particularly in relation to reports of treatment delays. This is due to the advent of a maximum waiting time standard of two weeks for people with FEP from referral to the start of treatment in an EIP service (NHS England, 2023). NHS England used a phased implementation of this standard since 2016 (NHS England, 2023), rising to a target that 95% of people with FEP were treated within these two weeks by 2024 (NHS England, 2023). This standard has substantially reduced waiting times for people with FEP in England; in 2023/24, 70% of people referred to services in England had this standard met (Royal College of Psychiatrists. State of the Nation Report, 2025), albeit falling short of the 95% target. Nevertheless, some service users may still experience delays to their treatment, which could harm their care and downstream outcomes.

## CONCLUSION

In our scoping review, we found that service users might experience harms when in EIP services. For those experiencing such harms these could lead to distress, feelings of abandonment or helplessness, medication side effects, and a mistrust of staff and services. These harms occurred across cultural, systemic and relational levels, which may act as a framework to guide service improvement to minimize harm to service users. We have made several recommendations to minimize harms in EIP care which could be evaluated, including trauma‐informed approaches, improved staff‐service user relationships, and improving advocacy, which was recommended by our lived experience advisors. We suggest that future research explicitly aims to appraise the full range of harms service users may experience against the potential beneficial experiences and outcomes that EIP services also provide.

## AUTHOR CONTRIBUTIONS

**Georgie Hudson:** Conceptualization; investigation; writing – original draft; methodology; writing – review and editing; formal analysis; project administration; software; data curation; visualization; validation. **India Francis‐Crossley:** Writing – review and editing; validation; data curation. **Craig S. Mackie:** Writing – review and editing. **Rachel Hiller:** Writing – review and editing; supervision; methodology. **James B. Kirkbride:** Methodology; writing – review and editing; supervision. **Claire Powell:** Conceptualization; supervision; methodology; writing – review and editing.

## CONFLICT OF INTEREST STATEMENT

None declared.

## Supporting information


Data S1.

## Data Availability

The data are available in OSF at https://osf.io/y76nw.
