# Supplementary material for: A scoping review of service user experiences of potential harm in early intervention in psychosis services in the UK
Source: Br J Clin Psychol. 2026 Apr 16;65(4):1015–39. doi: 10.1111/bjc.70057 (PMC13655988; doi:10.1111/bjc.70057)
Supplement: Supplementary file 1 [file BJC-65-1015-s001.docx]

Supplementary Material

PsychINFO & Embase Strategy

Group 1: Qualitative

1. EXP Qualitative Methods/
2. EXP qualitative Measures/
3. EXP interviews/
4. EXP content analysis/
5. Qualitative OR interview* OR “focus group*” OR ethnograph* OR phenomenology OR “grounded theory” OR “content analysis” OR “thematic analysis” OR “discourse analysis” OR “conversation analysis” OR “framework analysis” OR “narrative analysis” OR “mixed method*” OR “interpretative phenomenological analysis” OR “key informant” OR “participant observation” OR “appreciative inquiry”
6. 1 OR 2 OR 3 OR 4 OR 5

Group 2: Experiences

7. “lived experience”
8. (consumer* OR “service user*” OR client* OR patient OR “expert by experience” OR "experts by experience") ADJ2 (experience* OR satisfaction OR perception* OR perspective* OR narrative* OR view* OR opinion*)
9. 7 OR 8

Group 3: Early intervention

10. EXP early intervention/
11. (early OR first OR initial OR primary) ADJ2 (intervention* OR treatment* OR recognition OR detection OR admission* OR hospital* OR episode* OR breakdown* OR onset)
12. “first episode psychosis” OR FEP OR EIP OR EIS OR incidence OR “early psychosis intervention”
13. 10 OR 11 OR 12

Group 4: Psychosis

14. EXP Psychosis/
15. EXP Hallucinations/
16. Schizophreni* OR psychosis OR psychot* OR hallucin* OR delusion* OR paranoi* OR (voice ADJ1 hear*) OR “Negative symptom*” OR “Positive symptom*” OR “unusual belief*” OR “false belief*” OR “thought disorder”
17. 14 OR 15 OR 16

Overall

18. 6 AND 9 AND 13 AND 17
19. limit 18 to (english language and yr="2000 -Current")

MEDLINE Strategy

Group 1: Qualitative

1. EXP Qualitative Research/
2. EXP interview/
3. EXP Interview, Psychological/
4. Qualitative OR interview* OR “focus group*” OR ethnograph* OR phenomenology OR “grounded theory” OR “content analysis” OR “thematic analysis” OR “discourse analysis” OR “conversation analysis” OR “framework analysis” OR “narrative analysis” OR “mixed method*” OR “interpretative phenomenological analysis” OR “key informant” OR “participant observation” OR “appreciative inquiry”
5. 1 OR 2 OR 3 OR 4

Group 2: Experiences

6. “lived experience”
7. (consumer* OR “service user*” OR client* OR patient OR “expert by experience” OR "experts by experience") ADJ2 (experience* OR satisfaction OR perception* OR perspective* OR narrative* OR view* OR opinion*)
8. 6 OR 7

Group 3: Early intervention

9. (early OR first OR initial OR primary) ADJ2 (intervention* OR treatment* OR recognition OR detection OR admission* OR hospital* OR episode* OR breakdown* OR onset*)
10. “first episode psychosis” OR FEP OR EIP OR EIS OR incidence OR “early psychosis intervention”
11. 9 OR 10

Group 4: Psychosis

12. EXP Psychotic disorders/
13. EXP Hallucinations/
14. Schizophreni* OR psychosis OR psychot* OR hallucin* OR delusion* OR paranoi* OR (voice ADJ1 hear*) OR “Negative symptom*” OR “Positive symptom*” OR “unusual belief*” OR “false belief*” OR “thought disorder”
15. 12 OR 13 OR 14

Overall

16. 5 AND 8 AND 11 AND 15
17. limit 16 to (english language and yr="2000 -Current")

Web of Science Strategy

Group 1: Qualitative

1. Qualitative OR interview* OR “focus group*” OR ethnograph* OR phenomenology OR “grounded theory” OR “content analysis” OR “thematic analysis” OR “discourse analysis” OR “conversation analysis” OR “framework analysis” OR “narrative analysis” OR “mixed method*” OR “interpretative phenomenological analysis” OR “key informant” OR “participant observation” OR “appreciative inquiry”

Group 2: Experiences

2. “lived experience”
3. (consumer* OR “service user*” OR client* OR patient OR “expert by experience” OR "experts by experience") NEAR/2 (experience* OR satisfaction OR perception* OR perspective* OR narrative* OR view*)
4. 2 OR 3

Group 3: Early intervention

5. (early OR first OR initial OR primary) NEAR/2 (intervention* OR treatment* OR recognition OR detection OR admission* OR hospital* OR episode* OR breakdown* OR onset*)
6. “first episode psychosis” OR FEP OR EIP OR EIS OR incidence OR “early psychosis intervention”
7. 5 OR 6

Group 4: Psychosis

8. Schizophreni* OR psychosis OR psychot* OR hallucin* OR delusion* OR paranoi* OR (voice NEAR/2 hear*) OR “Negative symptom*” OR “Positive symptom*” OR “unusual belief*” OR “false belief*”

Overall

9. 1 AND 4 AND 7 AND 8
10. limit 9 to (english language and yr="2000 -Current")

Scopus Strategy

Group 1: Qualitative

1. Qualitative OR interview* OR “focus group*” OR ethnograph* OR phenomenology OR “grounded theory” OR “content analysis” OR “thematic analysis” OR “discourse analysis” OR “conversation analysis” OR “framework analysis” OR “narrative analysis” OR “mixed method*” OR “interpretative phenomenological analysis” OR “key informant” OR “participant observation” OR “appreciative inquiry”

Group 2: Experiences

2. “lived experience”
3. (consumer* OR “service user*” OR client* OR patient OR “expert by experience” OR "experts by experience") W/2 (experience* OR satisfaction OR perception* OR perspective* OR narrative* OR view*)
4. 2 OR 3

Group 3: Early intervention

5. (early OR first OR initial OR primary) W/2 (intervention* OR treatment* OR recognition OR detection OR admission* OR hospital* OR episode* OR breakdown* OR onset*)
6. “first episode psychosis” OR FEP OR EIP OR EIS OR incidence OR “early psychosis intervention”
7. 5 OR 6

Group 4: Psychosis

8. Schizophreni* OR psychosis OR psychot* OR hallucin* OR delusion* OR paranoi* OR (voice W/2 hear*) OR “Negative symptom*” OR “Positive symptom*” OR “unusual belief*” OR “false belief*”

Overall

9. 1 AND 4 AND 7 AND 8
10. limit 9 to (english language and yr="2000 -Current")

CINAHL Strategy

Group 1: Qualitative

1. (MH "Qualitative Studies+")
2. (MH "Interviews+")
3. Qualitative OR interview* OR “focus group*” OR ethnograph* OR phenomenology OR “grounded theory” OR “content analysis” OR “thematic analysis” OR “discourse analysis” OR “conversation analysis” OR “framework analysis” OR “narrative analysis” OR “mixed method*” OR “interpretative phenomenological analysis” OR “key informant” OR “participant observation” OR “appreciative inquiry”
4. 1 OR 2 OR 3

Group 2: Experiences

5. “lived experience”
6. (consumer* OR “service user*” OR client* OR patient OR “expert by experience” OR "experts by experience") N2 (experience* OR satisfaction OR perception* OR perspective* OR narrative* OR view* OR opinion*)
7. 5 OR 6

Group 3: Early intervention

8. (MH "Early Intervention+")
9. (early OR first OR initial OR primary) N2 (intervention* OR treatment* OR recognition OR detection OR admission* OR hospital* OR episode* OR breakdown* OR onset*)
10. “first episode psychosis” OR FEP OR EIP OR EIS OR incidence OR “early psychosis intervention”
11. 8 OR 9 OR 10

Group 4: Psychosis

12. (MH "Psychotic Disorders+")
13. (MH "Hallucinations+")
14. Schizophreni* OR psychosis OR psychot* OR hallucin* OR delusion* OR paranoi* OR (voice N2 hear*) OR “Negative symptom*” OR “Positive symptom*” OR “unusual belief*” OR “false belief*”
15. 12 OR 13 OR 14

Overall

4 AND 7 AND 11 AND 15
